# Cumulative Risk Assessment Toolbox: Methods and Approaches for the Practitioner

**DOI:** 10.1155/2013/310904

**Published:** 2013-05-09

**Authors:** Margaret M. MacDonell, Lynne A. Haroun, Linda K. Teuschler, Glenn E. Rice, Richard C. Hertzberg, James P. Butler, Young-Soo Chang, Shanna L. Clark, Alan P. Johns, Camarie S. Perry, Shannon S. Garcia, John H. Jacobi, Marcienne A. Scofield

**Affiliations:** ^1^Environmental Science Division, Argonne National Laboratory, Argonne, IL 60439, USA; ^2^ENVIRON International Corporation, Emeryville, CA 94608, USA; ^3^US Environmental Protection Agency, Office of Research and Development, National Center for Environmental Assessment, Cincinnati, OH 45268, USA; ^4^Biomathematics Consulting and Department of Environmental and Occupational Health, Emory University, Atlanta, GA 30322, USA; ^5^Synergy Toxicology, Boerne, TX 78006, USA; ^6^Baker Hughes, Tulsa, OK 74107, USA; ^7^ToxStrategies, Austin, TX 78759, USA; ^8^TEAM Integrated Engineering, Inc., San Antonio, TX 78216, USA

## Abstract

The historical approach to assessing health risks of environmental chemicals has been to evaluate them one at a time. In fact, we are exposed every day to a wide variety of chemicals and are increasingly aware of potential health implications. Although considerable progress has been made in the science underlying risk assessments for real-world exposures, implementation has lagged because many practitioners are unaware of methods and tools available to support these analyses. To address this issue, the US Environmental Protection Agency developed a toolbox of cumulative risk resources for contaminated sites, as part of a resource document that was published in 2007. This paper highlights information for nearly 80 resources from the toolbox and provides selected updates, with practical notes for cumulative risk applications. Resources are organized according to the main elements of the assessment process: (1) planning, scoping, and problem formulation; (2) environmental fate and transport; (3) exposure analysis extending to human factors; (4) toxicity analysis; and (5) risk and uncertainty characterization, including presentation of results. In addition to providing online access, plans for the toolbox include addressing nonchemical stressors and applications beyond contaminated sites and further strengthening resource accessibility to support evolving analyses for cumulative risk and sustainable communities.

## 1. Introduction

The public has become increasingly aware of the wide variety of chemicals present—not just in the environmental media to which they are exposed (such as air, water, and soil) but also in the food they eat and the products they use. As access to relevant information continues to grow, notably via the Internet, many communities have voiced concerns about health effects associated with the multiple chemicals in their daily lives. To address these concerns, many organizations have responded with approaches, guidelines, focused workshops, and illustrative applications to better assess cumulative risks. These organizations include the US Environmental Protection Agency (EPA), National Institute for Occupational Safety and Health (NIOSH), Agency for Toxic Substances and Disease Registry (ATSDR), World Health Organization (WHO), California Environmental Protection Agency (Cal/EPA), the Environmental Justice (EJ) community, and professional organizations such as the Society of Toxicology.

Cumulative risk assessment (CRA) explicitly considers the combined fate and effects of multiple contaminants from multiple sources through multiple exposure pathways. The goal of CRA is to address more realistic conditions than those addressed under the classic approach of the 1983 National Research Council (NRC) “red book” on *Risk Assessment in the Federal Government* [1], which agencies historically implemented by evaluating one chemical at a time. Reflecting advances in scientific knowledge since that time, which made more detailed and integrated analyses possible, EPA outlined its *Framework for Cumulative Risk Assessment* [2] in 2003 that considered joint exposure to multiple chemical, physical, and biological stressors. A number of organizations have published additional cumulative risk reports over the past decade [3–9]. Despite the increase in relevant analyses and reports, the translation of a more fully integrated approach to practice has lagged behind the science. With various groups and individual community members unaware of available tools that could be used to assess cumulative risk, explicit applications have been relatively modest.

The concept of a “cumulative risk toolbox” emerged soon after the EPA framework was published, during the early stages of developing a companion report to support evaluations at contaminated sites. The purpose of this CRA toolbox was to overcome the “awareness” hurdle and serve as a practical resource for cumulative risk assessors and other interested parties. Recognizing that cumulative risk encompasses many different kinds of stressors ranging from chemical and physical to biological and psychological ones, the CRA toolbox compilation that began in 2004 targeted a specific scope, namely, health risks from exposure to multiple anthropogenic chemicals as well as to elevated concentrations of chemicals that occur naturally. The main objectives of the CRA toolbox were to (1) consolidate resources for conducting cumulative risk assessments, (2) provide coverage for the main elements of the assessment process, and (3) offer practical notes to help assessors understand the strengths and limitations of a given approach or model for cumulative risk applications. 

The original CRA toolbox was published as an appendix to the 2007 Agency report, *Concepts, Methods, and Data Sources for Cumulative Health Risk Assessment of Multiple Chemicals, Exposures, and Effects: A Resource Document* [10]. It was recently reevaluated to determine if the descriptions and electronic web links for the individual entries were current or needed to be updated. This paper highlights relevant excerpts from the CRA toolbox with interpretive context for CRAs at contaminated sites, with updates as indicated; it also identifies several additional resources not included in the original CRA toolbox. 

## 2. Methods

The CRA toolbox was developed in three phases: (1) identification of information resources related to CRA, (2) assessment of their relevance to contaminated sites and facilities, and (3) categorization of the relevant resources to facilitate their use in analyses. In searching the scientific literature and other online materials for information related to CRA, the resources pursued included conceptual approaches, models, and other tools related to assessing health risks posed by multiple chemicals in multiple environmental media via multiple exposure routes. In addition, risk analysts from several agencies, universities, and the private sector were informally invited to identify methods and models they had found useful for assessing multiple contaminants, exposures, and effects as part of their environmental projects and programs. 

Some information evaluated in the second phase was designed to assess ecological effects, while other material addressed acute and shorter-term human exposures; however, the primary emphasis of the CRA toolbox was on approaches and tools for evaluating health effects from chronic to lifetime exposures. An example of this type of resource is the NIOSH approach for worker protection that accounts for exposures to chemical mixtures [11]. The resources considered less well suited than others to this type of application were screened out at this stage, as were resources that essentially duplicated content that had already been compiled. In the third phase, the resources selected for inclusion were collectively evaluated to identify common themes. The purpose of this last phase was to define a manageable set of categories to serve as the organizational structure of the CRA toolbox. This toolbox was not intended to be comprehensive; rather, it was developed to provide a suite of resources, including guidelines, approaches, and models, that could be applied to assess cumulative health risks associated with contaminated sites. For this reason, three basic documents guided the determination of the CRA toolbox categories: the 1983 NRC risk assessment paradigm [1], the 1989 EPA *Risk Assessment Guidance for Superfund* [12], and the 2003 EPA *Framework for Cumulative Risk* [2]. 

## 3. Results and Discussion

### 3.1. Illustrative Information Resources

The online searches and interactions with risk practitioners produced more than 100 methods and tools relevant to CRAs at contaminated sites. Not all explicitly target multiple contaminants, exposures, or effects. That is, many were developed for general risk assessment purposes, but they were found to be either directly useful or adaptable for population-specific CRAs, or the underlying approach was found to offer useful insights for such analyses. Many of the materials addressed one or two components of a CRA, while others addressed multiple elements or the overall concept. Although certain resources clearly consider multiple exposures to multiple chemicals, such as the standard Agency guidance for risk assessment at contaminated sites [12], only a fraction are explicitly defined as cumulative risk tools. Relatively few specifically focus on exposure groupings or joint toxicity, or on a given population group for CRA, such as children (e.g., see (4.1) in Table 4 and (6.7) in Table 6). Although many focus on contaminant sources or affected environmental media, as has been the historical practice, they can also be applied to more complex cumulative risk analyses. Similarly, various resources can be helpful for evaluating subsets of an overall cumulative risk assessment, such as cumulative hazards, threats, exposures, or impacts, including health impact analyses.

The resources compiled for the CRA toolbox are available on websites managed by a variety of organizations, including (1) federal agencies and institutes such as the EPA, US Nuclear Regulatory Commission, US Department of Energy (DOE), and US Department of Health and Human Services, including NIOSH and ATSDR within the Centers for Disease Control and Prevention (CDC), as well as the National Institutes of Health (NIH), including the National Institute of Environmental Health Sciences (NIEHS); (2) national scientific organizations such as the National Academies, including the National Academy of Sciences and National Research Council; (3) other federal entities such as the National Environmental Justice Advisory Council; (4) national organizations in other countries, including Canada (Health Canada, Environment Canada, and the Quebec worker protection institute IRSST, Institut de Recherche Robert-Sauvé en Santé et en Sécurité du Travail) and The Netherlands (Organization for Applied Scientific Research, Nederlandse Organisatie voor Toegepast Natuurwetenschappelijk Onderzoek (TNO) Nutrition and Food Institute); (5) international bodies, such as the European Commission and WHO; (6) private nonprofit organizations such as the International Institute of Indigenous Resource Management (IIIRM); (7) state agencies, such as the Cal/EPA Office of Environmental Health Hazard Assessment (OEHHA) and Air Resources Board (ARB); (8) industry organizations such as the American Chemistry Council (ACC); (9) scientific groups at universities and national laboratories; and (10) local community advisory boards at contaminated sites and facilities, such as site-specific, restoration, and citizen advisory boards (SSABs, RABs, and CABs).

### 3.2. CRA Toolbox Categories

**Figure 1:**
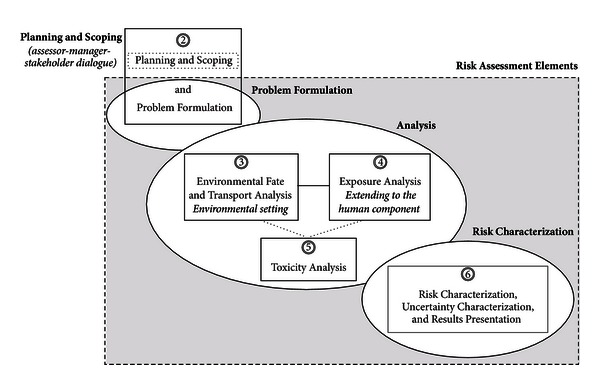
Relationships among the five CRA toolbox categories and key risk elements (*circles reflect table numbers*).

The CRA toolbox is divided into five sections as described in Table 1 and illustrated in Figure 1, with some sections further subdivided to identify specific content. (The resource count in Table 1 refers to the number of tools highlighted in tables for each topic.)

### 3.3. Resource Highlights

The resources highlighted in this section focus on those identified in the original CRA toolbox (which was developed in 2004), with limited updates. Several additional tools that include more recent content are noted in Section 3.5. While several tools apply to multiple categories, they are generally listed with the first CRA toolbox category for which they are particularly useful. (Note that planning and scoping for CRAs continues iteratively throughout the assessment process.) Each section begins with a topical introduction, followed by text bullets highlighting selected resources, and concluding with a table that summarizes the following for a larger number of entries: (1) title, author/organization, and access information (e.g., web link), where available; (2) purpose and scope; (3) remarks on application to CRA. Nearly 80 resources are summarized across the five tables combined, with additional resources highlighted in the accompanying text.

#### 3.3.1. Category 1: Resources for Planning, Scoping, and Problem Formulation (Table 2)

Topics addressed during planning, scoping, and problem formulation include the purpose and breadth of the assessment (considering multiple chemicals, population groups, exposures, and effects), the type of product needed from the assessment to inform a decision, the data to be collected and synthesized, the general assessment approach, and stakeholder involvement. CRAs can involve a very large number of potential combinations of chemicals and interactions inherent to the environmental setting. During this initial and iterative phase of the process, common questions include which chemicals are most likely to contribute significantly to risks, whether they might interact, and what the nature of those interactions could be. The Internet has become a valuable tool for promoting and enhancing stakeholder involvement, and successful programs often combine traditional methods (ranging from one-on-one to town hall meetings and printed newsletters or other information sent in regular mail) with electronic approaches to take advantage of the unique benefits of each. Selected resources that can be used to support planning, scoping, and problem formulation for CRAs, including stakeholder involvement, are presented in Table 2; several specific resources are highlighted below.

*Planning and Scoping.*  In 1997, EPA released its *Guidance on Cumulative Risk Assessment, Part 1, Planning and Scoping* ((2.1) in Table 2) that presented broad-based approaches that considered (1) multiple endpoints, sources, pathways, and routes of exposure; (2) community-based decision making; (3) flexibility in achieving goals; (4) case-specific responses; (5) all environmental media; and (6) holistic risk reduction. The companion *Lessons Learned on the Planning and Scoping of Environmental Risk Assessments *((2.2) in Table 2) followed in 2002, presenting case studies to illustrate organizing principles for CRAs. The following year, the Agency published its *Framework for Cumulative Risk Assessment* ((2.3) in Table 2) that outlines an umbrella structure for CRAs, identifies key issues, and defines common terms. Neither a procedural guide nor a regulatory requirement, the *Framework* document summarizes key elements of the CRA process as part of a flexible structure rather than identifying prescriptive protocols. Providing the basic information about important aspects of cumulative risk, that framework continues to serve as a key foundation for CRA reports. One example of a tool based on the framework is EJView ((2.4) in Table 2), a GIS-based module jointly designed by the EPA Offices of Environmental Information (OEI) and Environmental Justice (OEJ). Combining environmental, socioeconomic, and health indicators in statistical tables, this tool was initially developed to evaluate potential EJ issues. For community-based approaches, this and other ranking and prioritization tools can help identify the problems warranting consideration in a CRA. Note that although it is presented here within the planning/problem formulation stage to support front-end scoping, this and other such tools are also very useful for other phases of the CRA process, including risk characterization.

*Stakeholder Involvement.* A number of tools have been developed to support stakeholder involvement in CRAs, particularly for contaminated sites. Ranging from guidance for EPA's Superfund and EJ programs to project-specific field activities (see the second section of Table 2, (2.5) through (2.10)), these resources chronicle approaches taken to solicit inputs from multiple stakeholders and incorporate them into the assessment plans. A number of examples from DOE legacy waste sites reflect inputs of long-standing community advisory boards, from the DOE Savannah River Site in Georgia to the DOE Hanford Site in Washington. Practical insights can also be gained from the Risk Analysis, Communication, Evaluation, and Reduction (RACER) project at the DOE Los Alamos National Laboratory (LANL) in New Mexico. Led by the Risk Assessment Corporation (RAC) team, extensive stakeholder involvement has been a hallmark of that effort ((2.8) in Table 2).

*Data Quality Objectives.* The EPA has developed a series of guidance documents to help ensure that all data collection, including at contaminated sites, is appropriate for the intended use, which is particularly important for CRAs given the typical complexity of these analyses. These documents outline a systematic process for developing performance criteria to collect, evaluate, and use environmental data. Statistical and analytical tools underlie data quality objectives (DQOs), as highlighted in the last section of Table 2 ((2.11) through (2.17)).

#### 3.3.2. Category 2: Resources for Environmental Fate and Transport Analyses (Table 3)

A large number of tools have been developed to assess the environmental fate and transport of chemicals, and many of these can be used to support CRAs, as highlighted below and in Table 3. These tools include computer models available from the EPA Center for Subsurface Modeling Support (CSMoS), as well as resources for physicochemical constants and guidance for determining background concentrations in soil.

*Fate and Transport. *Risk assessments for contaminated sites and also for urban environments and other settings impacted by multiple pollutant sources commonly simulate the behavior of multiple chemicals in the environment because of the relatively high costs (in terms of both manpower and dollars) to conduct measurements. Hundreds of computer models have been developed to model contaminant fate and transport in the environment. Some are very general and conceptual, while others address specific media characteristics and setting conditions. The use and suitability of individual models vary widely depending on the project objectives and data required, so it is important for the selected model to be appropriate for the given evaluation. CSMoS is a key resource for these tools; the center maintains an online database of publicly available groundwater and vadose zone fate and transport models, a number of which are included in Table 3 (see entries (3.13)–(3.23)). Selected tools are also highlighted below. 

*Physicochemical Constants.* Several online resources provide information on the chemical, physical, and biological properties of substances, including industrial products and byproducts. Resources highlighted in the original toolbox include the EPA ChemBioFinder Database ((3.1) in Table 3) and Soil Screening Guidance ((3.2) in Table 3), which includes an extensive set of environmental and physical constants and parameters that can be used to model the fate and transport of chemicals in soil. The chemical-specific properties used to derive EPA Regional Screening Levels are also available online (see related discussion for Table 5). Information about these properties can be used to predict that chemicals will likely share a similar environmental fate to support exposure groupings for CRAs. 

*Background Concentrations.* Concentrations that represent natural background or ambient conditions are location specific and provide valuable context for assessing chemical fate and transport as well as incremental risk. The EPA has outlined an approach for characterizing background concentrations, including protocols for determining whether site measurements are statistically elevated, in *Guidelines for Characterizing Background Chemicals in Soil at Superfund Sites* ((3.3) in Table 3). Data on background concentrations of inorganic chemicals can be found in several sources that provide baseline context for community-based environmental risk assessments. Information sources include ATSDR toxicological profiles ((5.4) in Table 5) and technical reports from the US Geological Survey, as illustrated by a report on constituents of ambient surface soil in Chicago that was prepared in cooperation with the City of Chicago [13]. Similar regional data can be found in state-specific resource reports, such as from the Massachusetts Department of Environment [14] as well as the Texas Commission on Environmental Quality (TCEQ) [15], regarding background levels of polycyclic aromatic hydrocarbons (PAHs) and other constituents in soil. (Also, see related TCEQ entry (6.5) in Table 6.) 

*Vapor Intrusion. *Vapor intrusion can be an important exposure pathway for multiple chemicals when volatile organic compounds are in the subsurface (e.g., soil and groundwater) and can migrate to indoor air. Contributions from vapor intrusion are commonly combined with estimates from other indoor air pathways (e.g., inhalation of volatiles during showering) to quantify aggregate exposures and risks for single chemicals (e.g., benzene) and cumulative risks for groups of chemicals (e.g., chlorinated solvents). This pathway has historically been evaluated using a model based on the equation published by Johnson and Ettinger in 1991 [16]. The model is a one-dimensional spreadsheet that estimates convective and diffusive transport of chemical vapors to indoor air from sources near a building's perimeter. Attenuating factors such as biological degradation were not included in the original model, and the source was assumed to be infinite over the exposure duration assessed (e.g., 25 years for a commercial or industrial worker). The EPA provided a detailed description of this earlier vapor intrusion model in its draft guidance issued in 2002, and since that time, the Agency and others have continued to strengthen the modeling approach ((3.11) in Table 3). Following the early Johnson and Ettinger model, several US states have adopted simple equations based on this method to conduct screening evaluations of indoor air ((3.11) in Table 3). The indoor air concentrations calculated by these models across multiple chemicals can be combined to estimate cumulative exposures and corresponding risks.

#### 3.3.3. Category 3: Resources for Exposure Analysis (Table 4)

Many exposure models are well suited to assessing multiple chemicals by multiple routes, although this is generally performed by combining predictions made for individual chemicals. Tools range from relatively straightforward screening models to comprehensive multimedia models, as highlighted below and further illustrated in Table 4. Certain models also support other portions of the risk assessment process. For example, models for subsurface vapor migration described earlier are often tapped for multimedia exposure assessments because they consider both soil and groundwater inputs. In addition, several technical reports identify exposure factors, their bases, and parameters commonly used to estimate cumulative exposures. This category includes resources linked to fate and transport models, in some cases to account for the time lag between release and exposure considering the movement of chemicals from source to receptor. The analysis of changing chemical exposures over time is also an important concept for grouping chemicals, and models that incorporate this factor are included in Table 3 (e.g., see (3.7), (3.14), and (3.15)). 

*Exposure Factors.* Risk assessments commonly rely on exposure models to capture receptor-specific factors that influence chemical intakes. For example, factors that address exposure duration, time involved in certain activities, body weight and surface area, intake rates (e.g., inhalation, or ingestion of food, soil, or water), and many others parameters needed to estimate potential risks from multiple exposures are available from the EPA 2011 *Exposure Factors Handbook *and 2008 *Child-Specific Exposure Factors Handbook *(see (4.1) in Table 4). To support the evaluation of potentially vulnerable or susceptible subgroups, EPA's 1999 report on *Sociodemographic Data Used for Identifying Potentially Highly Exposed Populations* ((4.2) in Table 4) provides information to help identify subsets of the general population who may be at greater risk for negative health consequences, which can be incorporated into CRAs. An additional valuable resource is the National Human Exposure Assessment Survey (NHEXAS, (4.3) in Table 4), which was developed by EPA in the early 1990s to compile information on human exposures to chemicals at the community and regional scales, with an emphasis on associating these exposures with personal activities. The NHEXAS database is also noted in Table 4.

*Multipathway Releases and Exposures.* The 3MRA model ((4.4) in Table 4) is a multimedia, multipathway, multireceptor exposure and risk assessment model developed by EPA to assess releases from land-based waste management units. After simulating releases from disposal units, modules model fate and transport through the environment, estimate exposure to receptors, and calculate distributions of risks to receptors. The 3MRA methodology uses a Monte Carlo scheme to quantify uncertainty (e.g., from natural variability or based on selection of representative sites). The Exposure and Fate Assessment Screening Tool (E-FAST, (4.5) in Table 4) is another computer-based model that can provide screening-level estimates for general population, consumer, and environmental exposures to chemicals released to air, surface water, or landfills and those released from consumer products. Potential inhalation, dermal, and ingestion doses resulting from these releases are estimated, with the modeled concentrations and doses designed to reasonably overestimate exposures for use in screening-level assessments. States have also developed tools to assess exposures via the air pathway, such as California's Air Toxics “Hot Spots” Program that requires stationary air emission sources in the state to report the types and quantities of certain substances routinely released to air and also to estimate potential exposures to surrounding populations. A software package was developed to support these evaluations ((4.10) in Table 4). 

*Dietary Exposures.* The Dietary Exposure Potential Model (DEPM) ((4.12) in Table 4) estimates dietary exposure to multiple chemicals based on data from several national, government-sponsored food intake surveys and chemical residue monitoring programs. The DEPM includes recipes developed specifically for exposure analyses that link consumption survey data for prepared foods to the chemical residue information, which is normally reported for raw food ingredients, to estimate daily dietary exposure. The summary databases are aggregated in a way that allows the analyst to select appropriate demographic factors, such as age/sex groups, geographical regions, ethnic groups, and economic status. The model also includes modules for evaluating chemical exposures from residues, soil, and tap water. 

#### 3.3.4. Category 4: Resources for Toxicity Analyses (Table 5)

Resources that can be used to support toxicity analyses for CRAs are highlighted here and summarized in Table 5. Topics include (1) resources for toxicity reference values for various exposure routes and durations; (2) development of toxicity factors, including for whole mixtures; (3) identification of toxicity criteria for similar or surrogate compounds or mixtures to represent a mixture or its components; and (4) joint toxicity of the components of a mixture. 

*Toxicity Reference Values.* The EPA Integrated Risk Information System (IRIS) ((5.7) in Table 5) is a key source of information on chronic toxicity reference values, including reference doses (RfDs), reference concentrations (RfCs), and oral slope factors, unit risks, and corresponding risk-based concentrations [17] (see (5.7) in Table 5). Chronic toxicity values are also available in IRIS for certain mixtures (such as the RfC for diesel exhaust and cancer toxicity values for polychlorinated biphenyls). Note that if a standard toxicity value is not available in IRIS, a Provisional Peer-Reviewed Toxicity Value (PPRTV; derived for use in the Superfund Program) may be available ((5.8) in Table 5). PPRTVs are derived using the same methods, data sources, and EPA guidance used to derive IRIS values but undergo a comparatively more rapid scientific review [18]. If a relevant PPRTV is not available, some states have also developed selected toxicity values that may be found in summary tables of EPA Regional Screening Levels ((5.11) in Table 5). Recent IRIS assessments commonly include data on effects other than the critical effect used to derive a toxicity value; these effects (sometimes called secondary effects) can be used to at least qualitatively assess joint toxicity (usually via dose or response addition) of combined chemical exposures. The information provided in EPA's RfD arrays also could be used to support estimates of target organ toxicity doses based on secondary effects [19]. The ATSDR has developed toxicological profiles for many individual chemicals that identify the effects of the given chemical, as well as primary environmental and metabolic transformation products to support grouping by specific target organs and systems. A smaller set of interaction profiles has also been developed that assesses joint toxicity [3] (see (5.4) in Table 5). 

*Chemical Mixtures and Pesticides: Common Mode of Action.* In 2000, EPA updated its 1986 guidelines for chemical mixtures ((5.1) in Table 5). This supplemental guidance describes risk assessment approaches that depend on the type, nature, and quality of available data, and it includes equations, definitions, discussions of toxicological interactions and pharmacokinetic models, and approaches for assessing whole mixtures, surrogate mixtures, and individual mixture components. The whole-mixture discussion includes the derivation of whole-mixture toxicity values (RfDs, RfCs, cancer slope factors, and inhalation unit risks), as well as consideration of comparative potency and environmental transformations. The component discussion includes dose addition, the hazard index (HI), interaction-based HI, relative potency factors (RPFs), and response addition. 

In 2002, EPA published guidance for assessing the cumulative risk of pesticides with a common toxic mechanism to address requirements set forth in the Food Quality Protection Act of 1996 [20] (see (5.2) in Table 5); that guidance was updated in 2006 [21]. Note that *common mechanism* was interpreted by EPA as common mode of action [20]. The initial guidance considered potential exposures to 30 organophosphate pesticides via food, drinking water, and residential uses, and it applied methods to account for variable exposures per different ages, seasons, and geographic factors. 

EPA scientists in the National Center for Environmental Assessment collaborated with colleagues in the National Toxicological Research Center of the Food and Drug Administration to develop additional component approaches for assessing the cumulative risk posed by exposures to multiple chemicals by evaluating three scenarios [22, 23]. They explored a simple scenario in which it was certain that all chemicals being considered shared a common toxic mode of action, so a dose-additive approach could be applied. In the second scenario, modes of action for the chemicals in the mixture were known and could be used to divide the chemicals into independent mode-of-action subclasses; dose addition and response addition were then integrated to assess the risk. In the third scenario, the mode or modes of action were uncertain for the chemicals in the mixture, so a joint dose-response modeling procedure was developed that created a range of risk estimates. 

*Physiologically Based Models and Chemical Mixtures Toxicology Research.* Statistically based methods and computer tools that can model interactions and effects associated with multiple chemicals continue to be developed and refined. A main area of study involves applying physiologically based pharmacokinetic/pharmacodynamic (PBPK/PD) models to chemical mixtures. Reaction network modeling is an example of a computer-based approach that has been used in petroleum engineering to predict chemical reaction rates, products, and outcomes based on various statistical methods (including Monte Carlo-type analysis). A molecular-based model (BioMOL) was designed to use the reaction network modeling approach to predict effects of chemicals in complex biological systems [24].

Joint toxicology studies have also improved the understanding of potential health effects of chemical mixtures with different modes of action. For example, the TNO Nutrition and Food Research Institute of The Netherlands has evaluated the use of mechanistic models to describe interactions between mixture components expected to act by different modes of action. In a pilot study funded by the American Chemistry Council Long-Range Research Initiative (see (5.10) in Table 5), the TNO team applied PBPK models to assess possible toxicokinetic interactions between compounds in an applied mixture and compared those estimates to empirical dose-response modeling of observed pathological changes in the liver, blood, and kidney. The aim of such research is to develop and refine methods to be applied to other chemical mixtures. Other TNO studies have developed and applied statistical methods combining multivariate data analysis and modeling in *in vitro* and *in vivo* studies on various chemical mixtures such as petroleum hydrocarbons, aldehydes, food contaminants, industrial solvents, and mycotoxins [25, 26]. 

Similarly, NIH/NIEHS has sponsored studies on mixtures toxicology and environmental health, including as part of the National Toxicology Program (NTP), for which related reports and fact sheets are available from the NIEHS website [27]. A search engine on this website can be used to tap research and tools for specific applications, including those related to cumulative risk. NIEHS also publishes *Environmental Health Perspectives*, a monthly journal that often reports on studies relevant to chemical mixtures, with some issues and supplements entirely dedicated to mixtures. Also, NIH maintains the National Library of Medicine and other databases (see (5.9) in Table 5). In addition to the organizations highlighted in Table 5, others have also been assessing mixtures to support CRAs during the past decade. For example, the Health Canada Toxic Substances Research Initiative (TSRI) assessed cumulative effects of environmental toxics to both human and ecological receptors [28, 29]. Cal/EPA conducted a health assessment in the 1990s that focused on a representative complex mixture, diesel particulate matter (DPM) [30]. The scientific review panel for this study used the analyses of epidemiological data from workers to develop a unit risk estimate for diesel particulates, which was then used to derive an inhalation slope factor. This approach offered insights not only for assessments involving diesel exhaust but also for other chemical mixtures. 

*Benchmark Dose Software (BMDS) and Categorical Regression (CatReg).* The EPA developed the BMDS to fit mathematical models to toxicological dose-response data for a particular toxic effect ((5.6) in Table 5). The user evaluates results of this statistical software to select a benchmark dose (BMD) associated with a predetermined benchmark response (BMR), such as a 10% increase in the incidence of a particular lesion or a 10% decrease in body weight. A goal of the BMD approach is to define a point of departure to derive an RfD or RfC that is more independent of study design than the traditional method based on a single experimental dose, such as the no-observed-adverse-effect level (NOAEL). The HI uses RfDs or RfCs in a formula that is based on dose addition to scale the exposure levels in a mixture, producing an indicator of the extent of concern for toxicity. The BMD values used with dose addition could estimate a BMD for the mixture, allowing the mixture dose to be interpreted in terms of the risk of a particular effect. BMD modeling could also be applied to whole mixture dose-response data.

The categorical regression tool CatReg was developed to conduct dose-response modeling of data on diverse endpoints across multiple toxicological studies by categorizing effects into different severity levels, such as no-effect and adverse-effect levels ((5.3) in Table 5). The EPA has suggested that categorical regression results for a single chemical can yield benchmark doses (e.g., a 10% effective dose or ED_10_) or risk estimates that reflect the probability of observing a severity level of (nonspecific) response [10]. Once this is done for each component of a mixture, risk assessment methods for chemical mixtures such as the HI or response addition approach can then be applied to yield an indication of risk for the mixture. For CRAs, CatReg can be applied to evaluate grouped chemicals considering multiple effects. Therefore, this tool could be helpful for both the toxicity assessment and risk characterization components of an integrated risk analysis. 

*Risk-Based Screening Levels.* Risk-based screening concentrations have been developed for environmental media (including soil, drinking water, and air) by several organizations, including EPA. For example, EPA Regions 3, 6, and 9 previously developed risk-based concentrations (RBCs), medium-specific screening levels (MSSLs), and preliminary remediation goals (PRGs), respectively; these similar values were subsequently combined to produce regional screening levels (RSLs) for assessing contaminated sites ((5.11) in Table 5). These screening values can be used to narrow the focus of assessments on key contributors to risks; they are based on conservative default assumptions for exposure and environmental parameters, and they reflect toxicity values from IRIS and other sources (e.g., Cal/EPA). 

#### 3.3.5. Category 5: Resources to Characterize Risk and Uncertainty and Present Results (Table 6)

Many assumptions are made when assessing health risks from environmental exposures to multiple chemicals. Thus, it is important for the risk estimates and associated uncertainties to be well characterized and clearly presented so this information can be interpreted appropriately to guide sound decisions. This final phase of the CRA process has come to rely on issue-specific summaries and graphical tools to display statistical and spatial information, as highlighted here and in Table 6. 

*Spatial Analysis and Decision Assistance Tool (SADA).* The SADA tool was jointly developed by the EPA, US Nuclear Regulatory Commission, and University of Tennessee as an integrated software package to support human and ecological CRAs (see (6.1) of Table 6). Like many other tools (including the RSLs), the human health module of SADA includes equations from the standard Superfund guidance [12] and accommodates different land use scenarios and exposure pathways. These can be combined to estimate overall exposures for the selected receptors. This tool emphasizes the spatial distribution of contaminant data, and individual modules cover visualization, geospatial analysis, statistical analysis, sampling design, and decision analysis. Outputs can be tabular or graphical, and they can be used to identify where estimated risks exceed target values. Although the input data for these pathways can be tailored to reflect site-specific conditions, interactions are not considered. 

*Probabilistic Methods and Tools.* Risk assessments commonly present human health risks as single-point estimates (e.g., 1 × 10^−5^) in accordance with standard guidance for contaminated sites [12]. Such estimates provide little information about the underlying uncertainty or variability. For example, Monte Carlo simulation tools can be used to consider the effect of uncertainty and variability, with results approximating a full range of reasonably possible outcomes that are typically plotted as graphs (e.g., frequency distributions) or tabulated. While these probabilistic approaches have not yet been widely implemented in environmental health risk analyses for contaminated sites, such simulations can help assessors represent uncertainty and variability in the risk results (see (6.7) in Table 6). Extensive research in probabilistic analysis relevant to CRAs has been conducted at a number of universities, including North Carolina State University (C. Frey and colleagues) and the University of Washington (A. Cullen and colleagues). These evolving tools incorporate distributions of parameter values that may be more appropriate for a given assessment than point estimates, and they are particularly well suited for CRAs. 

*Community-Based Air Pathway Modeling and Other Joint Exposure-Risk Resources. *The EPA Region 6 Regional Air Impact Modeling Initiative (RAIMI) was originally designed as a GIS-based system that could tap a number of emissions data sources to assess potential impacts at the community level ((6.3) in Table 6). This system also supports source attribution analyses, and early findings indicated that a small number of sources were responsible for most of the impact. Initially implemented at a pilot scale that focused on risks from direct inhalation exposures, RAMI was subsequently expanded to assess indirect exposures resulting from airborne releases, thus increasing the relevance for additional CRA applications. Tools identified with the exposure assessment phase that consider the spatial scale of various impacts (e.g., (4.7) in Table 4) can also be valuable resources for the risk characterization phase of CRAs. Similarly, disease registries that provide context for exposure assessments (see (4.15) in Table 4) also serve as resources for risk characterization by presenting health data that can be considered in concert with modeled or measured chemical data to assess potential influences of multiple exposures (including population-specific or location-specific patterns) and to calibrate risk models.

### 3.4. Review Findings

This reevaluation of the original toolbox (as described in EPA's 2007 report, *Concepts, Methods, and Data Sources for Cumulative Health Risk Assessment of Multiple Chemicals, Exposures, and Effects: A Resource Document* [10]) found that only half the web links were still valid, as most websites and models have been updated since the original toolbox was published. However, most information is still available in some form, and only a few web pages no longer exist. The web links presented in this overview of the original toolbox reflect the updated web addresses as of early 2013.

The review of the original toolbox also found that many compilations, approaches, and models have been updated, and additional resources are now available online. For example, a targeted search produced more than twice the number of resources reflected in the original toolbox. Additional organizations represented in this ongoing update of the CRA toolbox include the US Army Corps of Engineers, US Geological Survey, and US Department of Agriculture.

Both new and updated models can be found via a number of EPA (and other) websites, including the Center for Exposure Assessment Modeling (CEAM), National Center for Environmental Assessment (NCEA), and National Exposure Research Laboratory (NERL), as well as the Council for Environmental Regulatory Modeling (CERM). Most of these additional resources focus on the broad topic of exposure, including environmental fate and transport, but some address toxicity and risk characterization resources. Selected examples are provided in the following section.

### 3.5. Selected CRA Resources in addition to Those Listed in the Original Toolbox

Several cumulative risk meetings held within the last several years have produced compilations in a manner similar to the effort undertaken for the original toolbox in 2004 to support the EPA CRA resource document (which was published in 2007). These include workshops organized by EPA that involved external scientists, as well as an internal EPA workshop that convened cumulative risk experts and EPA Program and Regional project leads in July 2009 to share insights from recent and ongoing applications. The models and other resources compiled from some of these meetings are available in the scientific literature and online [31–33]. Furthermore, the NRC published two studies relevant to cumulative risk [7, 8], one in 2008 that focused on a specific class of chemicals (*Phthalates and Cumulative Risk Assessment: The Task Ahead*), and the other in 2009 that focused on how current risk analysis approaches could be improved (*Science and Decisions: Advancing Risk Assessment*). In addition, after an extensive process of collaborative workshops and soliciting input from interested parties, in late 2010 Cal/EPA OEHHA released its report on *Cumulative Impacts: Building a Scientific Foundation* [6]. 

For the exposure category, a key resource not included in the original toolbox is the CDC's National Health and Nutrition Examination Survey (NHANES) database. This database contains information on the health and nutritional status of adults and children, and it is unique in its incorporation of data from both interviews and physical examinations. NHANES is also a source of information on the distribution of some contaminant concentrations in human tissues drawn from samples collected in the US population. Valuable research findings have been made possible by this unique database, including recent evaluations of cadmium exposures and effects in children that inform risk assessments for contaminated products from overseas [34]. Information about the NHANES program and research publications can be found online via the CDC website [35]. 

For the toxicity category, in addition to the recent online availability of the EPA PPRTVs [18], the IRIS database [17] now provides additional toxicity information that can be used to assess chemical mixtures. For example, information is included from recent toxicological reviews that evaluate common modes of action and common target organs and systems, which can be considered in evaluating secondary effects and establishing toxicity groupings for CRAs. NCEA has also developed a new database of the toxicity literature for a number of chemicals, called Health and Environmental Research Online (HERO), which provides information underlying recent risk-related EPA analyses and can also be mined to support CRAs [36].

The International Toxicity Estimates for Risk Assessment (ITER) database is another toxicity resource [37], which was created and is maintained by the nonprofit scientific organization Toxicology Excellence for Risk Assessment (TERA). Its tabulated data include toxicity values and cancer classifications for more than 600 chemicals, with synopses that explain data differences and web links to the source organizations for further information.

Progress also continues on the international front, spanning each key element of a CRA. In 2009, the WHO released its draft report for public review, *Risk Assessment of Combined Exposures to Multiple Chemicals: A WHO/IPCS Framework* [4]. Also that year, the European Commission moved forward with its ongoing initiative, *NoMiracle (Novel Methods for Integrated Risk Assessment of Cumulative Stressors in Europe)*, including making a toolbox available to support these analyses [9].

Only a few of the recent resources have been highlighted here. Many more continue to be developed and refined to support CRAs, as the science evolves to meet the demands of the broader community for more realistic assessments that can guide effective risk management decisions. Resource toolboxes such as this one can support more integrated compilations to help address the need for increased awareness and access to practical approaches for cumulative risk assessors. CRAs that tap resources in this toolbox—which extend from EPA's cumulative risk framework and resource document to conceptual models, exposure factor handbooks, and toxicity databases, together with census data, specific fate models, community involvement processes, and new visualization tools—are being reflected in the growing literature on this topic (e.g., see [32, 38–44]).

## 4. Conclusions

A number of existing methods and tools can be applied to assess cumulative risks for contaminated sites, and progress continues in developing and refining such resources. Most of the tools in the original CRA toolbox have been updated, and others have become available. Nevertheless, explicit CRA applications are not yet widespread as issues related to general awareness and integration of relevant tools persist. Meanwhile, programs continue to evolve that are well suited to CRA approaches, which could help enhance the understanding of, and guide measures to address, combined stressors before they become a problem. Thus, the need exists for a broadly accessible toolbox that can serve as a practical foundation for cumulative risk practitioners across agencies, academia, and the private sector, as well as for interested members of the general public. Plans designed to address this need include the following:

- Strengthen the online accessibility of CRA toolbox resources;
- Provide additional practical context from case studies; and
- Incorporate further tools that extend to nonchemical stressors and other applications, to address emerging themes including sustainable communities.

## Figures and Tables

**Table 1:** Five categories and example resources from the CRA toolbox tables.

Topic	Resource count	Resource highlights
Planning, scoping, and problem formulation	18	Includes resources for the main category shown as well as for the subcategories of stakeholder involvement and data quality; it also includes a geographic assessment tool for Environmental Justice (EJ) applications and other prioritization tools, together with project-specific examples.

Environmental fate and transport analysis	24	Includes resources for physicochemical constants, guidance for determining background concentrations in soil, soil screening, and selected models available from the EPA Center for Subsurface Modeling Support (CSMoS).

Exposure analysis (extending to human factors)	15	Includes resources linked to fate and transport that also extend to human activities, such as technical information and models to predict dispersion of airborne contaminants released from industrial facilities and waste sites. It also includes resources for exposure factors, sociodemographic data, and the human exposure assessment database.

Toxicity analysis	12	Includes resources for toxicity reference values for specific exposure routes and durations, criteria for determining similar chemicals or surrogates for assessing mixtures, and approaches for assessing joint toxicity.

Risk and uncertainty characterization and presentation of results	10	Includes a cumulative risk index analysis, a spatial analysis and decision assistance tool, and other geographic information system (GIS)-based tools, as well as probabilistic approaches and a method for developing an environmental load profile.

**Table 2:** Selected resources for planning, scoping, and problem formulation.

Resource and access	Purpose and scope	Cumulative risk remarks
*Resources for planning, scoping, and problem formulation*		

(2.1) Guidance on Cumulative Risk Assessment—Part 1, Planning and Scoping (EPA); http://www.epa.gov/OSA/spc/pdfs/cumrisk2.pdf	Published in 1997, this guidance outlines EPA policy for planning and scoping for CRAs. The guidance directs each office of the EPA to take into account cumulative risk issues in scoping and planning major risk assessments and to consider a broader scope that integrates multiple sources, effects, pathways, stressors, and populations for cumulative risk analyses in all cases for which relevant data are available. This guidance also includes discussion pertaining to community-based decision making, flexibility in achieving goals, case-specific responses, a focus on all environmental media, and holistic reduction of risk.	Identifies four key steps for planning and scoping: determine overall purpose and risk management objectives for assessment; determine scope, problem statement, participants and resources; determine risk dimensions and technical elements that may be evaluated; formulate a technical approach including a conceptual model and analysis plan for the assessment.

(2.2) Lessons Learned on Planning and Scoping for Environmental Risk Assessments (EPA); http://www.epa.gov/osa/spc/ pdfs/handbook.pdf	Published in 2002, this report provides feedback to EPA scientists and managers regarding EPA's experiences with planning and scoping as the first step in conducting environmental assessments. It is intended to reinforce the importance of formal planning and dialogue prior to conducting complex cumulative assessments and to provide case studies and “lessons learned” for planning.	Provides information and feedback from the Part 1 planning guidance that offer insights for designing and conducting CRAs.

(2.3) Framework for Cumulative Risk Assessment (EPA); http://www.epa.gov/raf/publications/framework-cra.htm	Published in 2003, the document provides a flexible framework for CRAs. It identifies the basic elements of the process, describes a number of technical and coordination issues, and defines terms. This framework has served as a foundation for the CRAs developed since its publication.	Defines general structure and components of CRAs; provides the groundwork for scoping context considered in this paper.

(2.4) EJView (EPA); http://epamap14.epa.gov/ejmap/entry.html	Jointly developed by the EPA Office of Environmental Information and Office of Environmental Justice, EJView is a GIS-based module that can be used to guide front-end scoping of CRAs. It combines environmental, socioeconomic, and demographic data and health indicators in statistical tables, as well as providing facility-level data.	This tool can help identify problems to be assessed in a CRA. Although presented here within the planning/problem formulation phase, this tool is also useful for other phases, including risk characterization.

*Resources for stakeholder involvement*		

(2.5) Superfund Community Involvement Handbook, Appendix A: Superfund Community Involvement Requirements (EPA); http://www.epa.gov/superfund/community/involvement.htm; http://www.epa.gov/superfund/community/cag/pdfs/ci_handbook.pdf	Superfund guidance on suggested community involvement structure, communications, and approach. For contaminated sites, the lead agency informs the public of the availability of technical assistance grants (TAGs). The TAG is a grant program that provides funds for citizen groups to hire independent technical advisors to help them understand and comment on technical decisions regarding Superfund cleanup actions. (This is now part of a broader community involvement toolkit.)	Developed for the Superfund program; with cross-cutting information about community involvement, including forming community advisory groups (CAGs), this resource is also useful for CRAs at contaminated sites.

(2.6) Community Air Screening How-To Manual (EPA); http://www.epa.gov/oppt/cahp/pubs/howto.htm	Explains how to form a partnership, clarify goals, develop a detailed local source inventory, and use a risk-based process to identify priorities and develop options for risk reduction. Developed by the EPA's Office of Pollution Prevention and Toxics based on the Baltimore (Maryland) approach.	Presents a step-by-step process a community can follow to form a partnership to access technical expertise, identify and inventory local sources of air pollutants, review these sources to identify known hazards that might pose a health risk to the community, and set priorities and develop a plan for making improvements. Covers only the air pathway.

(2.7) Hanford Site (DOE), Hanford Advisory Board (HAB), Public Involvement Resources and Calendar; http://www.hanford.gov/page.cfm/hab, http://www.hanford.gov/public/calendar/	HAB was established to provide recommendations and advice to DOE, EPA, and the State of Washington's Department of Ecology on a number of issues related to cleanup of the Hanford site. Among its activities, the HAB created a calendar for public involvement to list upcoming meetings and other events at which input from affected parties and stakeholders was encouraged. A comment response tracking system was also developed to coordinate issues identified by stakeholders during iterative planning and scoping, throughout the assessment process, and to track followups.	The HAB mission language, online tools, and other information can serve as examples for other CRA projects.

(2.8) Los Alamos National Laboratory (LANL) (DOE), Risk Assessment Corporation (RAC), Risk Analysis, Communication, Evaluation, and Reduction (RACER) project; http://www.racteam.com/racer.html	The Risk Assessment Corporation (RAC) team developed an open process for assessing cumulative risks at LANL and for creating a decision analysis framework for risk reduction, with guidance for participation and an integrated database (with data from multiple collecting organizations) to support risk analyses. Stakeholder participation was actively sought in both open progress meetings and one-on-one meetings held in various settings; the Internet was also used, to announce activities and availability of draft documents for stakeholder review and to solicit inputs. Objectives were to develop (1) a process for extensive stakeholder involvement in risk assessment and decision-making processes for LANL; (2) a method for estimating current human health risks and ecological impacts using available data on chemicals and radionuclides measured in environmental media; (3) a method for implementing a comprehensive risk-informed decision analysis framework, including a prospective risk and ecological impact assessment to guide long-term management of risks and ecological impacts; (4) a consistent approach for compiling, using, and updating data to support the risk assessment and decision-making processes. The RACER project has also involved local schools in science projects, inviting the public to provide input to exposure scenarios.	Insights for cumulative assessments can be found in the RAC guidelines for stakeholder involvement, open survey questions, plans for soliciting (in various venues) and summarizing inputs to guide the assessment, and suggestions for pursuing grants for ongoing stakeholder involvement (aimed to be administered through an independent group), as well as other plans and products that can be found on the project website.

(2.9) Savannah River Site (DOE), Citizens Advisory Board (CAB); http://www.srs.gov/general/outreach/srs-cab	A CAB was created to facilitate public outreach for the DOE Savannah River Site, consisting of 25 individuals who reflect the cultural diversity of the local population. The CAB provides advice and recommendations to DOE, EPA, and the South Carolina Department of Health and Environmental Control on environmental remediation, waste management, and related issues. Regular meetings and public comment sessions were kept open to the public.	Recommendations and information on workshops published on this website can offer insights for similar projects.

(2.10) Weldon Spring Site (DOE), Partners in Education; http://www.lm.doe.gov/Weldon/CPAR_WSSRAP_Update_Jun92.pdf; http://www.lm.doe.gov/Weldon/10_23_2002summary.pdf	A scientific educational partnership established more than 20 years ago at the Weldon Spring Site in Missouri promoted community involvement in evolving evaluations for this DOE legacy waste site, toward ultimately supporting site cleanup plans. An open door policy with the community translated to weekly meetings during certain periods.	This early project illustrated the essential role of the community in developing and implementing a CRA for a legacy waste site.

*Resources for guiding data quality*		

(2.11) Guidance on Systematic Planning Using the Data Quality Objectives Process (EPA, QA/G-4); http://www.epa.gov/quality/qs-docs/g4-final.pdf	Published in 2006, this guidance outlines a systematic planning process for collecting environmental data. Designed to help analysts ensure that data are collected for a specific purpose, it includes the approach for determining which chemicals to evaluate or test for, in which media, and at what locations, as well as detection limits.	Recommended planning process when environmental data are used to select between two opposing conditions, useful for CRAs. The focus is on the (cumulative risk) questions to be answered, while maintaining awareness of the appropriate statistical techniques that should be considered to produce scientifically defensible results.

(2.12) Software (EPA, QA/G-4D); http://www.epa.gov/quality/qs-docs/g4d-final.pdf	Computer-based software for determining the feasibility of DQOs using a systematic process. Calculates the appropriate number of environmental samples required to statistically answer whether soil or water concentrations are above or below a risk-based level; can be used to estimate sampling costs.	General analytical guidance can be applied to multiple media and multiple contaminants. Could be adapted to support chemical grouping.

(2.13) Guidance on Choosing a Sampling Design for Environmental Data Collection (EPA, QA/G5S); http://www.epa.gov/quality/qs-docs/g5s-final.pdf	Guidance on applying standard statistical sampling designs (such as simple random sampling) and more advanced sampling designs (such as ranked set sampling and adaptive cluster sampling) to environmental applications.	Can be useful to identify colocated contaminants to support grouping for a CRA at a contaminated site or situation.

(2.14) Guidance for Quality Assurance Project Plans for Modeling (EPA, QA/G-5M); http://www.epa.gov/quality/qs-docs/g5m-final.pdf	General guidance for developing quality assurance project plans (QAPPs) for modeling projects.	Can be useful for CRAs, particularly where air or groundwater models are needed to extrapolate small data sets to the site or community level.

(2.15) Guidance on Environmental Data Verification and Data Validation (EPA, QA/G-8); http://www.epa.gov/quality/qs-docs/g8-final.pdf	Guidance to help organizations verify and validate data. Applying this to laboratory analytical data allows analysts to understand uncertainties associated with concentration measurements (which impacts assessment results).	Useful for determining appropriate data for chemicals to be evaluated in a CRA; important with regard to results, especially when using conservative screening approaches.

(2.16) Data Quality Assessment: A Reviewer's Guide (EPA, QA/G-9R); http://www.epa.gov/quality/qs-docs/g9r-final.pdf	Describes procedures and methods for ensuring sound data are used in the risk assessment. Identifies tools for reviewing DQOs and sampling design, reviewing preliminary data, selecting statistical tests to summarize and analyze data, verifying the assumptions of the statistical test, and performing calculations.	Can indicate differences in statistical robustness that might affect data combinations for chemical grouping and selection of representative concentrations.

(2.17) Data Quality Assessment: Statistical Methods for Practitioners (EPA, QA/G-9S); http://www.epa.gov/quality/qs-docs/g9s-final.pdf	Same as (2.16).	Same as (2.16); for example, if some data were collected according to DQOs established with decision error feasibility trials while other data were collected under another program that required fewer samples, care would be warranted when combining these data.

**Table 3:** Selected resources for evaluating environmental fate and transport.

Resource and access	Purpose and scope	Cumulative risk remarks
(3.1) ChemBioFinder Database (private, linked via EPA at: http://www.epa.gov/oppt/sf/tools/measured.htm); http://chemfinder.cambridgesoft.com/	An online, EPA-linked search engine that provides access to information on the chemical, physical, and biological properties of a large number of chemicals. Developed by CambridgeSoft, this tool can search per the chemical's common name, brand name, Chemical Abstract Service (CAS) number, chemical formula, or other designations, including chemical structure.	Can also be useful to indicate common characteristics to support chemical grouping (e.g., by soil-water partition coefficient (*K*_*d*_) for exposure analyses, or considering biological properties to support toxicity screening).

(3.2) Soil Screening Guidance (EPA), and Supplemental Guidance for Developing Soil Screening Levels for Superfund Sites (EPA); http://www.epa.gov/superfund/health/ conmedia/soil/introtbd.htm; http://www.epa.gov/superfund/health /conmedia/soil/index.htm	This guidance was published in 1996, with updates continuing through the 2002 supplement; it includes an extensive set of environmental and physical constants and parameters that can be used to model the fate and transport of chemicals in soil and to develop risk-based soil screening levels (SSLs) to protect human health. Tables of chemical-specific constants include organic carbon partition coefficient (*K*_oc_), soil-water partition coefficient (*K*_*d*_), and water and air diffusivity constants (*D*_*w*_ and *D*_*i*_, resp.), as well as default values for such parameters as fraction of organic carbon in soil (*f*_oc_), dry soil bulk density (*ρ*_*b*_), and water-filled soil porosity (*θ*_*w*_), to support the evaluation of fate and transport. The primary goal is to provide simple screening information and a method for developing site-specific levels considering soil and groundwater; although presented in this section, it is also considered relevant to Table 4 for exposure-based screening.	Developed for use at contaminated sites on the National Priorities List, the concepts can be extended to other sites and situations. It presents both detailed models and generic SSLs that can be used to quickly (and conservatively) assess what areas or pathways might warrant more detailed analyses. The guidance includes tables of chemical-specific constants such as the *K*_oc_, *K*_*d*_, *D*_*w*_, and *D*_*i*_, as well as default values for parameters like *f*_oc_, *ρ*_*b*_, and *θ*_*w*_, to support analyses of fate and transport that can guide fate-based exposure groupings for CRAs.

(3.3) Guidance for Comparing Background and Chemical Concentrations in Soil for CERCLA Sites (EPA); http://www.epa.gov/oswer/riskassessment/ pdf/background.pdf	Published in 2002, this guidance outlines statistical methods for characterizing background concentrations of chemicals at contaminated sites. Developed for both human and ecological risk assessors as well as decision makers.	This guidance explicitly acknowledges the important role of background concentrations in communicating cumulative risks associated with contaminated sites and indicates that cumulative risk considers all exposure pathways and the chemicals associated with them.

(3.4) SBAT, Soil BioAccessibiity Tool (EPA); http://www.epa.gov/superfund//programs/ aml/tech/news/sbat.htm	Tool for estimating bioaccessibility of arsenic and chromium from soil on abandoned mine lands and implications for bioavailability (following ingestion). Results indicate that iron and manganese oxides can oxidize arsenic (III to V), and that organic matter and ferrous minerals reduce chromium (from VI to III), possibly reducing toxicities from oral exposure. Sequestration is enhanced by contact time (indicating less accessibility of metals from aged soils).	Provides context for fate of these combined metals in soil, highlighting specific factors to be measured or otherwise evaluated to produce a more realistic and practical site-specific assessment; these include predicted bioavailability following intake (notably specific physical and chemical properties of the soil).

(3.5) SESOIL, SEasonal SOIL compartment model (in the public domain although updated versions are available from RockWare, Inc.); http://www.rockware.com/; http://www.epa.gov/opptintr/exposure/pubs/gems.htm	SESOIL is a one-dimensional (1-D) vertical transport screening-level model for the unsaturated (vadose) zone that can be used to simulate the fate of contaminants in soil to support site-specific cleanup objectives. Simulates natural attenuation based on diffusion, adsorption, volatilization, biodegradation, cation exchange, and hydrolysis. The model can evaluate one chemical at a time; it does not predict interactions in environmental media.	Results can indicate how far a contaminant plume could migrate; predicted concentrations can be compared to media-specific standards and can be used to estimate single-chemical risks based on standard default exposure parameters, locations, and times. The location- and time-specific predictions for single chemicals can be overlain to support grouping decisions for a cumulative assessment.

(3.6) Summers model (as for (3.5)); http://www.seview.com/	Screening-level leachate code that estimates groundwater concentrations based on mixing. Simulates dilution of soil in groundwater. The model can evaluate one chemical at a time; it does not predict interactions in environmental media.	Same as (3.5) for SESOIL (and (3.7) for AT123D).

(3.7) AT123D, Analytical Transient 1-, 2- and 3-Dimensional Simulation of Waste Transport in the Aquifer System (EPA and private); http://www.scisoftware.com/; http://www.epa.gov/opptintr/exposure/ pubs/gems.htm	Generalized three-dimensional (3-D) groundwater transport and fate model; processes simulated include advection, dispersion, adsorption, and biodegradation as a first-order decay process. Transport can be simulated over 10,000 years. When linked with SESOIL, the model can simulate up to 1,000 years of contaminant migration. It can evaluate one chemical at a time (including radionuclides), and it can also evaluate heat (as a physical stressor); it does not predict interactions in environmental media.	Same as (3.5) and (3.6). This model can evaluate single chemicals, including radionuclides, and it can also evaluate heat (a physical stressor).

(3.8) MODFLOW, with many iterations/updates (USGS) http://water.usgs.gov/nrp/gwsoftware/modflow.html (Note Visual MODFLOW is available for a fee from the developer)	This widely used model numerically solves the 3-D groundwater flow equation for a porous medium by using a finite-difference method. Visual MODFLOW output is graphic, including 2-D and 3-D maps. Designed to model flow, it can evaluate one chemical at a time (information is input by the user); it does not predict interactions in environmental media.	Results can indicate how far a contaminant plume could migrate; predicted concentrations can be compared to media-specific standards and can be used to estimate single-chemical risks based on standard default exposure parameters, locations, and times. Location- and time-specific predictions for single chemicals can be overlain to support grouping decisions for a cumulative assessment.

(3.9) MULKOM codes, including TMVOC (and predecessor T2VOC) (Lawrence Berkeley Laboratory, DOE); http://www-esd.lbl.gov/TOUGH2	Three-dimensional, three-phase flow of water, air, and volatile organic compounds (VOCs) in saturated and unsaturated (vadose) zones to support remediation evaluations such as for soil vapor extraction. TMVOC can address more than one volatile organic (e.g., to model a spill of fuel hydrocarbons or solvents).	Similar to MODFLOW (see (3.8)), but it can address a mixture of VOCs. Like the other models, this set depends heavily on extensive site setting characterization for results to be meaningful; it can be difficult to get the data needed for all parameters.

(3.10) MT3D (links to MODFLOW); http://www.ess.co.at/ECOSIM/MANUAL/mt3d.html	Three-dimensional transport model for simulating advection, dispersion, and chemical reactions in groundwater systems; it assumes first-order decay and addresses one chemical at a time.	Chemical reactions can be addressed with a loss term (chemical data must be input by the user), but the degradation product is not tracked. Depends heavily on extensive site characterization; it can be difficult to get the data needed for all parameters.

(3.11) Guidance for Evaluating Vapor Intrusion into Buildings (EPA); http://www.epa.gov/oswer/vaporintrusion; state example: http://www.envirogroup.com/links.php; application: http://www.deq.louisiana.gov/portal/ Portals/0/RemediationServices/RPform_5340.pdf	Provides a model to estimate convective and diffusive transport of chemical vapors to indoor air. Could offer insights for situations where indoor air exposures are a concern. More than half the states also provide simplified equations for screening chemicals via the vapor intrusion pathway. For an indication of states, see the second web link. Example application context is provided from the Louisiana Department of Environmental Quality (LDEQ) via the third web link.	Model output can be used to support CRAs, as concentrations of multiple chemicals can be evaluated simultaneously.

(3.12) Risk Assessment Protocols for Hazardous Waste Combustion Facilities (EPA); http://www.epa.gov/osw/hazard/ tsd/td/combust/risk.htm; http://www.epa.gov/osw/hazard/ tsd/td/combust/ecorisk.htm	In 1998, EPA Region 6 identified a need for guidance that consolidated information from earlier EPA documents and state environmental agency reports, to provide an integrated set of procedures for conducting site-specific combustion risk assessments addressing multiple sources and exposure scenarios. Two documents were prepared, the 1999 Screening Level Ecological Risk Assessment Protocol for Hazardous Waste Combustion Facilities (SLERAP) and the 2005 Human Health Risk Assessment Protocol for Hazardous Waste Combustion Facilities (HHRAP). The objectives were to (1) apply the best available methods for evaluating risks to human health and the environment from operations of hazardous waste combustion units and (2) develop repeatable and documented methods for consistency and equity in permitting decisions. In addition to methods for evaluating multimedia, multipathway risks, the second document contains information on chemical, physical, and environmental properties of many chemicals, for use in modeling environmental fate and transport and exposure.	Provides methods for evaluating multimedia, multipathway risks. Volume II contains information and data on the physicochemical and environmental properties of many chemicals, which can be used to model environmental fate and transport and exposure. This information could be used to predict which chemicals are likely to share a similar fate in the environment, to support exposure groupings for CRAs.

*Information on the following models is available via compilations on EPA websites (including http://www.epa.gov/ada/csmos/ and http://www.epa.gov/esd/databases/datahome.htm); therefore, individual links are not provided in this section of the table.*		

(3.13) 2DFATMIC and 3DFATMIC	Simulates the subsurface flow, transport, and fate of contaminants that are undergoing chemical and/or biological transformations. Applicable to transient conditions in both saturated and unsaturated zones. Results can indicate how far a plume may migrate.	Predicted concentrations can be compared to media-specific standards to assess single-chemical risks using exposure parameters, locations, and times. The model can evaluate one chemical at a time; it does not predict interactions in environmental media. Location- and time-specific predictions for single chemicals can be overlain to support grouping decisions for a cumulative assessment.

(3.14) BIOCHLOR	Screening model that simulates remediation by natural attenuation of dissolved solvents at sites with chlorinated solvents. Can be used to simulate solute transport without decay and solute transport with biodegradation modeled as a sequential first-order process within one or two different reaction zones.	Same as (3.13) and (3.15).

(3.15) BIOPLUME II and BIOPLUME III	Model 2-D contaminant transport under the influence of oxygen-limited biodegradation (BIOPLUME II) and under the influence of oxygen, nitrate, iron, sulfate, and methanogenic biodegradation (BIOPLUME III). Model advection, dispersion, sorption, biodegradation (aerobic and anaerobic), and reaeration (BIOPLUME II) through instantaneous, first order, zero order, or Monod kinetics (BIOPLUME III). BIOPLUME III was developed primarily for modeling the natural attenuation of organic contaminants in groundwater; it is particularly useful at petroleum-contaminated sites.	Same as (3.13) and (3.15).

(3.16) BIOSCREEN	Screening-level groundwater transport model that simulates the natural attenuation of dissolved-phase hydrocarbons. It is based on the Domenico analytical contaminant transport model and can simulate natural attenuation based on advection, dispersion, adsorption, and biological decay. It estimates plume migration to evaluate risk at specific locations and times. (Selected model comparisons indicated that concentrations may be underestimated compared with AT123D and MODFLOW/MT3D.)	Predicted concentrations can be compared to media-specific standards and can be used to estimate single-chemical risks based on standard default exposure parameters, locations, and times. The model can evaluate one chemical at a time; it does not predict interactions in environmental media. Location- and time-specific predictions for single chemicals can be overlain to support grouping decisions for a cumulative assessment.

(3.17) CHEMFLO	Simulates 1-D water and chemical movement in the vadose zone. Models advection, dispersion, first-order decay, and linear sorption. Results can indicate how far a plume will migrate.	Same as (3.16).

(3.18) GEOEAS, Geostatistical Environmental Assessment Software	Enables geostatistical analysis of spatially correlated data. Can perform basic statistics and scatter plots/linear and nonlinear estimation (kriging).	Same as (3.16).

(3.19) GEOPACK	Comprehensive package for geostatistical analyses of spatially correlated data. Can perform basic statistics, variography, and linear and nonlinear estimation (kriging).	Same as (3.16).

(3.20) HSSM, Hydrocarbon Spill Screening Model	Can simulate light nonaqueous phase liquid (LNAPL) flow and transport from the ground surface to the water table; radial spreading of the LNAPL phase at the water table; dissolution and aquifer transport of the chemical. It is 1-D in the vadose zone, radial in the capillary fringe, and provides a 2-D vertically averaged analytical solution of the advection-dispersion equation in the saturated zone. (It is available in Spanish.)	Predicted concentrations can be compared to media-specific standards and used to estimate single-chemical risks based on exposure parameters, locations, and times. The model can evaluate one chemical at a time; it does not predict interactions in environmental media. Location- and time-specific predictions for single chemicals can be overlain for CRA groupings.

(3.21) PESTAN, Pesticide Analytical Model	Vadose zone modeling of the transport of organic pesticides. Models advection, dispersion, first-order decay, and linear sorption. Results can indicate how far a contaminant plume will migrate.	Same as (3.20).

(3.22) STF, Soil Transport and Fate Database	Provides data on the behavior of organic and a few inorganic chemicals in soil. (EPA review was designed to verify data accuracy; the information is believed to be accurate, but EPA does not make any claim regarding data accuracy and is not responsible for its use.)	This general-use tool can be used to evaluate the physicochemical properties of environmental contaminants for CRAs. The focus is one chemical at a time; interactions are not addressed.

(3.23) UTCHEM	Three-dimensional model that simulates aqueous phase and nonaqueous phase liquid (NAPL) movement in the subsurface. It can address multiple phases, dissolution, and/or mobilization by nondilute remedial fluids, chemical and microbiological transformations (including temperature dependence of geochemical reactions), and changes in fluid properties as a site is remediated.	This general-use tool can be applied to evaluate environmental contaminants for CRAs. It can be interesting when used to assess cumulative risk because NAPL is commonly a complex mixture itself and can be present in multiple phases, which are assessed by the model.

**Table 4:** Additional resources for evaluating exposure.

Resource and access	Purpose and scope	Cumulative risk remarks
(4.1) Exposure Factors Handbook (EPA); http://cfpub.epa.gov/ncea/risk/ recordisplay.cfm?deid=236252 (this 2011 handbook updated the 1997 document; highlights are also available, at http://cfpub.epa.gov/ncea/risk/ recordisplay.cfm?deid=221023); child-specific handbook: (published in 2008): http://cfpub.epa.gov/ncea/risk/ recordisplay.cfm?deid=199243	Provides extensive values and underlying bases for many factors that affect exposures. Examples include exposure duration, frequency, surface area, inhalation rate per activity level, and age/gender, as well as ingestion rates (including for incidental soil ingestion and by food type) based on age and gender. Because children can exhibit different exposure patterns to environmental toxicants than adults, the EPA published the *Child-Specific Exposure Factors Handbook* in 2008 to provide a summary of available statistical data on various factors assessing children's exposures.	Compendia of values for exposure parameters that can be reviewed to determine those most appropriate for a given site/setting, for adults and children. Can be used to assess multiple pathways and activities/intake rates for exposures to multiple chemicals.

(4.2) Sociodemographic Data Used for Identifying Potentially Highly Exposed Populations (EPA); http://cfpub.epa.gov/ncea/cfm/recordisplay.cfm?deid=22562	Setting-specific social and demographic characteristics can cause various subgroups to incur higher exposures than the general population. Published in 1999, this report provides information to help identify those population subgroups; it includes information related to activity patterns (how time is spent), microenvironments (where time is spent), and other data such as gender, race, age, and economic status. Fact Finder searches and returns data from this document.	Can be used to guide the identification and characterization of subgroups within the general population who could be at risk for higher contaminant exposures and related effects, to be addressed in a CRA.

(4.3) NHEXAS, National Human Exposure Assessment Survey (EPA); http://www.epa.gov/heasd/edrb/nhexas.html; HEDS, Human Exposure Database System; http://www.epa.gov/heds/	The EPA Office of Research and Development conducted the NHEXAS survey in the 1990s to assess U.S. exposures to chemicals in concert with their activities.	This extensive set of exposure data linked to activity patterns can be used to support CRAs, including providing insights into potentially vulnerable subpopulations.

(4.4) 3MRA (Center for Exposure Assessment Modeling, CEAM) (EPA); http://www.epa.gov/ceampubl/mmedia/3mra/index.htm	Developed for screening-level exposure and risk assessments for multiple media, multiple pathways, and multiple receptors, for potential human and ecological health risks from chronic exposures to chemicals released from land-based waste management units containing listed waste streams. Site based, it was intended for national-scale application to generate risk- based standards (e.g., levels to exit from hazardous waste regulation); it evaluates human and ecological receptors and captures uncertainty and variability in risk estimates. (Ecological exposure and risk focus on population effects related to key species within habitats found in the proximity of sites.)	Can quantify exposure via multiple pathways after a simulated release. Human receptors include adult/child residents, home gardeners, beef and dairy farmers, and recreational fishers. Pathways include inhalation of outdoor air and indoor air while showering, ingestion of drinking water, and ingestion of farming products and fish.

(4.5) E-FAST, Exposure and Fate Assessment Screening Tool (EPA); http://www.epa.gov/oppt/exposure/pubs/efastdl.htm	Provides screening-level estimates for general population, consumer, and environmental exposures to concentrations of chemicals released to air, surface water, and landfills and released from consumer products. It estimates potential inhalation, dermal and ingestion doses, and the modeled concentrations and doses are designed to reasonably overestimate exposures for use in screening-level assessments.	Default exposure parameters are available, but the use of site-specific values is recommended. Can predict exposure concentrations for comparison to media-specific standards.

(4.6) FRAMES, Framework for Risk Analysis in Multimedia Environmental Systems (DOE Pacific Northwest National Laboratory, in support of EPA); http://www.epa.gov/athens/research/modeling/3mra.html	Integrated software system to conduct screening-level assessments of health and ecological risks for hazardous waste identification rule (HWIR) chemicals from land-based waste management units.	Can be applied to conduct health and ecological screening of multiple chemicals for disposal facilities.

(4.7) TRACI, Tool for the Reduction and Assessment of Chemical and Other Environmental Impacts (EPA); http://www.epa.gov/nrmrl/std/traci/traci.html	TRACI is an impact assessment tool for evaluating multiple chemical-impact and resource-use categories to analyze various study designs. Impacts that can be modeled include ozone depletion, global warming, acidification, eutrophication, photochemical smog, cancer risk and noncancer health effects, human health criteria, ecotoxicity, fossil fuel use, land use, and water use. The program includes quantitative data on human carcinogenicity and noncarcinogenicity (based on human toxicity potentials), acidification, smog formation, and eutrophication. The model uses a probabilistic approach to determine spatial scale(s) for other impact categories such as acidification, smog formation, eutrophication, and land use.	Can be used to model and compare exposures to multiple chemicals and health risks associated with different projects. For example, it can graphically analyze the reduction in risk projected from one implementation design versus another. This tool is also relevant to risk characterization (Table 6).

(4.8) SCRAM, Support Center for Regulatory Atmospheric Modeling (EPA), includes links for air quality models, applications, and tools; http://www.epa.gov/ttn/scram/	Provides descriptions and documentation for different types of air quality models, information on modeling tools, and support for existing models. Also provides links to relevant workshops, conferences, reports, journal articles, and websites with further information about atmospheric and air quality models and monitors.	Good source of models, guidance, and other information useful for CRAs that involve air quality monitoring.

(4.9) Technology Transfer Network (TTN), CHIEF, Clearinghouse for Inventories and Emissions Factors (EPA); http://www.epa.gov/ttn/chief/	EPA resource of tools to support air pathway analyses. The TTN maintains a Clearinghouse for Inventories and Emission Factors (CHIEF) that links to a number of helpful technical documents on methods and data for constructing emissions inventories, including the Handbook for Criteria Pollutant Inventory Development: A Beginner's Guide for Point and Area Sources, Handbook for Air Toxics Emission Inventory Development, Volume I: Stationary Sources, and Compilation of Air Pollutant Emission Factors.	Source for many tools used to assess emissions and dispersion of contaminants released to air. For some cases (notably for metals, including radionuclides), unit particulate emissions can be used to scale to source concentrations in order to estimate airborne and deposited contaminant concentrations.

(4.10) HARP, Hotspots Analysis and Reporting Program Tool (California Air Resources Board, CARB); http://www.arb.ca.gov/toxics/ harp/downloads.htm#2	Software package for facility emissions inventory databases; prioritize facilities for management; model atmospheric dispersion of chemicals from one or multiple facilities using EPA models; calculate cancer and noncancer (acute and chronic) health impacts using Cal/EPA guidance; use point estimates or data distributions of exposures to calculate inhalation and multipathway risks; perform stochastic health risk analyses; calculate potential health effects for individual receptors, population exposures, cumulative impacts for one or multiple facilities and one or multiple pollutants, and potential health effects using ground-level concentrations; present results as tables and isopleth maps.	Designed to address multiple sources, pollutants, concentrations, and exposure pathways to estimate cumulative health effects. Also relevant to risk characterization (Table 6), results can be printed, added to reports, or input to a GIS.

(4.11) CalTOX Model; http://www.dtsc.ca.gov/AssessingRisk/caltox.cfm	Spreadsheet-based model that relates the concentration of a chemical in soil to the risk of an adverse health effect for a person living or working on or near a site. Defaults are available, but site-specific values are recommended. It estimates the chemical concentration in the exposure media of breathing zone air, drinking water, food, and soil that people inhale, ingest, and dermally contact, and uses the standard equations found in RAGS (EPA 1989) to estimate exposure and risk.	Can be used to assess multiple exposures; it has tended to be more for research than practical applications. It can predict exposure concentrations that can be compared to media-specific standards and used to estimate single-chemical risks, which could then be overlain for CRAs.

(4.12) DEPM, Dietary Exposure Potential Model (EPA); http://www.epa.gov/nerlcwww/depm.html	Estimates dietary exposures to multiple chemicals based on data from several national, government-sponsored food intake surveys and chemical residue monitoring programs. Includes recipes developed specifically for exposure analyses that link consumption survey data for prepared foods to chemical residue information, which is normally reported for raw food ingredients, to estimate daily dietary exposure. The summary databases are aggregated in a way that allows the analyst to select appropriate demographic factors, such as age/gender groups, geographical regions, ethnic groups, and economic status. Includes modules for evaluating exposures from residues, soil, and tap water.	Can be used to assess exposures to multiple chemicals from ingesting food and tap water; it could potentially provide context for ambient exposures in the area of a site.

(4.13) All-Ages Lead Model (EPA); http://cfpub.epa.gov/ncea/cfm/ recordisplay.cfm?deid=139314; supporting technical data and related methods and models are at http://www.epa.gov/superfund/ health/contaminants/lead/products.htm; related documents including interim soil lead guidance for CERCLA sites and RCRA corrective action facilities are also available: http://www.epa.gov/superfund/ lead/products/oswerdir.pdf	EPA model used to predict lead concentrations in body tissues and organs for a hypothetical individual based on a simulated lifetime of lead exposure, extrapolated to a population of similarly exposed individuals. Rather than external dose, most health effects data for lead are based on blood lead concentration which is an integrated measure of internal dose, reflecting total exposure from all sources (e.g., both site-related and background sources for Superfund sites). Both the EPA and Cal/EPA Department of Toxic Substances Control (DTSC) have developed models to estimate blood lead concentrations from exposures to lead from various media, including soil, water, air, and food. In addition to its tool for assessing exposures to children (IEUBK, integrated exposure uptake and biokinetic model), the EPA also developed a further set of models for evaluating lead exposures and risks for nonresidential adults (the all-ages model).	Useful for evaluating the impact of multiple sources of lead by multiple routes. Results could potentially be combined with risks estimated for certain other contaminants if interactions with lead are known to occur (e.g., see ATSDR interaction profiles, (5.4) in Table 5).

(4.14) NIOSH NORA Mixed Exposures program; http://www.cdc.gov/niosh/nora/	Provides technical and support information on projects involving mixed exposures in the workplace. National Occupational Research Agenda (NORA) program identified a number of research areas for NIOSH that addressed mixed occupational exposures, with an aim of protecting individuals in the workplace from exposures to multiple chemicals. The website for the mixed exposures team provides links to related studies, as well as information on how to join a listserv group to discuss topics related to mixed exposures.	Information resource for mixtures in the workplace; scientific knowledge developed through this effort can offer insights for assessing the combined effects of chemicals at contaminated sites, occupational settings, and other scenarios involving multiple chemicals.

(4.15) National Cancer Registry (CDC); http://www.cdc.gov/cancer/dcpc/data/ (this website includes links to various state registries); U.S. census data; http://www.census.gov	A number of health registry databases contain information on various diseases, conditions, and other health-related data, including cancer, asthma, birth defects, and blood lead levels. Organizations such as the CDC and others maintain these databases to allow these data to be evaluated in concert with modeled or measured chemical exposure data to correlate potential influences of multiple exposures and to calibrate risk models. For example, the CDC national registry of cancer cases includes cancer type and target tissue, as well as demographic and location information. Many state and local government health departments and other health organizations also maintain disease and condition registries to monitor trends over time; determine patterns in various populations; guide planning and evaluation of control programs; help set priorities for allocating health resources; advance clinical, epidemiologic, and health services research; provide information for a national database of cancer incidence. Other government resources can also be used to indicate vulnerable population groups who might be at increased risk, such as data from the U.S. Census Bureau.	Data could be used to indicate key community health concerns or for an exploratory investigation of a certain disease or condition that might increase the vulnerability of certain people who could be exposed to a given chemical. However, the links to diseases from environmental exposures or directly to environmental pollutants as causal or contributing factors are not usually clear. This tool is also directly relevant to risk characterization (Table 6).

**Table 5:** Selected resources for evaluating toxicity.

Resource and access	Purpose and scope	Cumulative risk remarks
(5.1) Supplementary Guidance for Conducting Health Risk Assessment of Chemical Mixtures (EPA); http://cfpub.epa.gov/ncea/cfm/recordisplay.cfm?deid=20533	Published in 2000, this EPA guidance supplements the EPA's 1986 guidelines for chemical mixtures and describes risk assessment approaches that depend on the type, nature, and quality of available data. The report presents approaches for assessing whole mixtures, surrogate mixtures and individual mixture components, including equations, definitions, and the theory behind dose addition, response addition, toxicological interactions, and the concept of sufficient similarity among whole mixtures. Guidance is given on how to practically use whole-mixture methods to develop a whole-mixture reference dose (RfD), reference concentration (RfC), and slope factor, and to assess comparative potency and environmental transformations. Guidance is also provided for using component-based methods, including the hazard index (HI); interaction-based HI; relative potency factors (RPFs); response addition.	Presents more detailed information on considerations and quantitative methods for assessing risks posed by exposures to environmental mixtures.

(5.2) Relative Potency Factors for Pesticide Mixtures, Biostatistical Analyses of Joint Dose- Response; http://cfpub.epa.gov/ncea/cfm/recordisplay.cfm?deid=66273	In response to requirements of the Food Quality Protection Act of 1996, the EPA prepared a technical report that presents research and methodologies for developing RPFs that can be used to assess cumulative risks from exposures to mixtures such as organophosphate pesticides, dioxins, and polychlorinated biphenyls (PCBs). The document presents three scenarios for which biostatistical methods for toxicity assessment can be used, including dose addition (for simple cases where common modes of toxicity are present), integration of dose and response addition (for cases where toxicities are independent), and joint dose-response modeling (for cases where the mode of action is uncertain).	Provides information that can be used to assess cumulative risks for sites contaminated with organophosphate pesticides and other organic compounds, such as dioxins and PCBs.

(5.3) CatReg, Categorical Regression (EPA); http://cfpub.epa.gov/ncea/cfm/recordisplay.cfm?deid=18162	This categorical regression model was developed for meta-analyses of toxicology data. The approach could be useful for evaluating different types of data to assess potential cumulative health risks.	Can be used to evaluate multiple effects within a chemical grouping (e.g., as grouped by target organ or system) and can also be used as a tool to support the estimate of potential health effect (e.g., hazard index) from multiple-route exposures.

(5.4) Toxicological Profiles (ATSDR); http://www.atsdr.cdc.gov/toxprofiles/index.asp; and Interaction Profiles for Toxic Substances; http://www.atsdr.cdc.gov/interactionprofiles/index.asp	Toxicological profiles exist for many chemicals, including some mixtures; they summarize data on sources and uses; physicochemical properties, environmental fate, and environmental levels; toxicity, including environmental and metabolic transformation products on specific target organs; critical effects, secondary organs and systems. ATSDR also prepared guidance for mixtures that outline an assessment approach, as well as interaction profiles for whole mixtures and selected combinations of individual chemicals with toxic interactions (often evaluated in pairs). These profiles include directions of interactions with confidence indicators by organ/system. Initial combinations are (1) arsenic, cadmium, chromium, and lead; (2) benzene, toluene, ethylbenzene, and xylene; (3) lead, manganese, zinc, and copper; (4) cyanide, fluoride, nitrate, and uranium; (5) cesium, cobalt, PCBs, strontium, and trichloroethylene; (6) 1,1,1-trichloroethane, 1,1-dichloroethane, trichloroethylene, and tetrachloroethylene; (7) arsenic, hydrazines, jet fuels, strontium-90, and trichloroethylene.	Some profiles address mixtures (e.g., PCBs). These reports can be useful for identifying endpoint-specific effects to support CRAs; the toxicity data organized by organ/system can be used to determine at what levels joint toxicity could be a factor, as an initial step to guide pursuit of the primary literature. Information is included for secondary effects (those occurring at doses higher than that corresponding to the most sensitive, or critical, effect), which can also support toxicity groupings for CRAs.

(5.5) Risk assessment guidelines (EPA); http://cfpub.epa.gov/ncea/cfm/recordisplay.cfm?deid=55907	Guidelines exist for carcinogens, chemical mixtures, ecology, neurotoxicity, reproductive toxicity, exposure assessment, developmental toxicity, and mutagenicity; these were developed to support risk evaluations based on recommendations from the National Academy of Sciences.	Outlines approaches and data that provide context for assessing mixtures and multiple endpoints. Can be used to guide toxicity groupings for CRAs.

(5.6) BMDS, Benchmark Dose Software (EPA); http://www.epa.gov/ncea/bmds/index.html	Designed to fit mathematical models to dose-response data so results allow the selection of a benchmark dose (BMD) associated with a predetermined benchmark response (BMR), such as a 10% increase in the incidence of a particular lesion or a 10% decrease in body weight.	BMD values used with dose addition could support estimation of a BMD for a mixture. For toxicity endpoints described by RfDs and RfCs, this approach would provide a risk-based dose associated with a particular effect.

(5.7) IRIS, Integrated Risk Information System (EPA); http://www.epa.gov/iris	Key source of chronic toxicity information and standard toxicity values including RfDs and RfCs, cancer slope factors unit risks and corresponding risk-based concentrations; it includes information for more than 500 chemicals. Combined with exposure information, these data can be used to characterize health risks from exposure to individual chemicals across multiple routes (where reference values are available). The toxicity values and information on target tissues included in IRIS summaries and technical support documents (TSDs) can be used in CRAs to identify chemicals that can exert primary as well as secondary effects on similar target tissues or systems. That is, although chemical interactions other than addition are not quantifiable using toxicity criteria from IRIS, the information in the accompanying technical evaluations can be used to qualitatively assess the nature and magnitude of certain interactions, and the ATSDR interaction profiles and the primary literature can be pursued for additional information.	Toxicity values address some chemical mixtures (e.g., PCBs, toxaphene, and others); target organ information can be used to group chemicals for CRAs, for example, to identify those exerting primary and secondary effects on common tissues or systems. Interactions other than addition are not quantifiable using these toxicity criteria; however, the nature and magnitude of some interactions could be predicted using the information provided, notably in the TSDs. The toxicity values can be used to estimate collective noncancer effects and cancer risks by summing, assuming additivity. Age-dependent adjustment factors can be applied as indicated in TSDs when estimating risks for sensitive subpopulations, assumed to incur childhood exposures (to age 16).

(5.8) PPRTV (Provisional Peer-Reviewed Toxicity Value) database (EPA); http://hhpprtv.ornl.gov	The PPRTV database is similar to IRIS in serving as a source of toxicity values, notably to address chemicals and exposure durations for which an IRIS value is not available, and a need for a provisional value has been identified.	Chemical mixtures for which PPRTVs are available include complex mixtures of aliphatic and aromatic hydrocarbons, midrange aliphatic hydrocarbon streams, and xylenes.

(5.9) TOXNET/HSDB, MEDLINE, PubMed, other databases (NIH); via http://www.nih.gov; for example, TOXNET/HSDB; http://toxnet.nlm.nih.gov/hsdb.htm	NIH sponsors and maintains several databases for toxicology and environmental health applications, including TOXNET and the Hazardous Substances Data Bank (HSDB), Haz-Map (occupational health database), PubMed, and MEDLINE, with links to biomedical journals. These contain thousands of entries for single chemicals and also include data for a substantial number of mixtures (such as PCBs, PAHs, coal tar, crude oil, and oil dispersants).	Useful source of peer-reviewed information that can be used for toxicity groupings to support CRAs. Although listed with toxicity tools, these databases also contain information to support exposure/fate groupings. The databases are expected to reflect further content relevant to cumulative risk as those data become available from ongoing research.

(5.10) LRI, Long-Range Research Initiative; http://www.uslri.org/; http://lri.americanchemistry.com/	Industry-funded scientific program included a cumulative risk focus area. Sponsored by the American Chemistry Council (ACC), research in this area emphasized assessment methods and toxicity studies for mixtures.	Research results could offer insights for CRAs at contaminated sites, including regarding joint toxicity.

(5.11) RSLs, Risk-Based Regional Screening Levels (EPA); http://www.epa.gov/reg3hwmd/risk/human/ rb-concentration_table/index.htm	RSLs for environmental media (soil, drinking water, and air) are based on specified risk levels, using conservative assumptions and established toxicity values primarily developed by EPA, as supplemented by other organizations (e.g., Cal/EPA). The RSLs were harmonized in 2008, combining the Region 3 risk-based concentrations (RBCs), Region 6 medium-specific screening levels (MSSLs), and Region 9 preliminary remediation goals (PRGs).	Emphasis is on multiple pathways and chemical concentrations rather than target organs or effects. Although not explicitly for CRAs, this tool includes a suite of equations that can be used to assess multiple pathways then combine results, and the screening basis can help focus a CRA on those chemicals likely to contribute substantially to overall risks.

(5.12) RESRAD, RESidual RADioactivity (DOE Argonne National Laboratory); http://www.ead.anl.gov/resrad	The original RESRAD code was designed to guide radiological cleanup criteria for contaminated sites and assess doses and risks from residual radionuclides. Sister codes cover chemical contaminants to support a combined evaluation of risks and hazard indices at sites with radionuclides and chemicals. The code includes a screening groundwater model and links to an air dispersion model; it also includes a probabilistic module. The toxicity values provided include radiological risk coefficients. Results can be presented in graphs and tables.	Can be used to assess doses and risks associated with radioactively (and chemically) contaminated facilities. Accounts for radioactive decay but not environmental transformation to address changes over time; produces risks and HIs summed across contaminants and pathways; does not address toxic interactions. Can conduct a probabilistic analysis and assess sensitivity, so this is also relevant for risk characterization (Table 6).

(5.13) VEMPire, Valeur d'Exposition Moyenne Pondérée (time-weighted average, worker exposure level), database (IRSST); http://www.irsst.qc.ca/en/-tool-vempire-5-5-version.html	Database for airborne chemicals commonly found in the workplace and at many contaminated sites. Addresses Canadian occupational standards (many are the same as U.S. standards), toxicokinetics, target organs, effect levels, and mode of action where available. The database also includes a calculation tool that allows up to 10 chemicals to be assessed at a time, comparing the concentration of interest to the occupational standard to produce a sum of ratios, assuming additivity as the default approach.	Source of useful inhalation toxicity data for a large number of chemicals. This tool could be used to organize chemicals by target organ and effect; exposure levels can be divided by reference levels (occupational standards), with an option for calculating a sum of ratios for 10 chemicals, assuming additivity. This approach could presumably be supplemented to account for interactions if/where known.

(5.14) Pesticides: Health and Safety, Common Mechanism Groups; Cumulative Exposure and Risk Assessment; http://www.epa.gov/oppsrrd1/ cumulative/common_mech_groups.htm	Identifies health information to assess pesticide groups that share common mechanisms of toxic action, with links for quantitative approaches (e.g., RPF values) and qualitative approaches (e.g., analysis of mode of action). The pesticide groups evaluated include organophosphates, triazines, n-methyl carbamates, and chloroacetanilides.	Can be used to assess index chemical-equivalent doses and risks associated with specific pesticide groups that share a common toxic mode of action.

**Table 6:** Selected resources for characterizing risk and uncertainty and presenting results.

Resource and access	Purpose and scope	Cumulative risk remarks
(6.1) SADA (Spatial Analysis and Decision Assistance); http://www.tiem.utk.edu/~sada/	Integrated software with flexible land use scenarios and exposure pathways. Emphasizes spatial distribution of contaminant data; modules cover visualization, geospatial analysis, statistical analysis, sampling design, and decision analysis. Outputs can be tabular or graphical. Can address both health and ecological risk to support integrated decisions.	Useful for cumulative risk assessments; can combine pathways to assess overall exposures and summed risks and HIs for receptors of interest. Input data can reflect site-specific conditions; interactions are not considered.

(6.2) RAIMI, Regional Air Impact Modeling Initiative (EPA); http://www.epa.gov/region6/6en/raimi/index.htm	Risk-based prioritization tool developed by Region 6 to support regional risk-based prioritization at the community level from exposures to multiple airborne contaminants from multiple sources via multiple exposure pathways. Designed to support cross-program analyses. Includes Risk-MAP, to estimate health risks from exposures to chemical emissions over large areas.	Assesses multiple contaminants and multiple sources for EPA programs, for air contaminants. Designed to consider source-specific and contaminant-specific contributions to cumulative exposures associated with the air pathway.

(6.3) Environmental Load Profile (EPA); http://www.epa.gov/region2/ej/guidelines.htm	Compares indicators of well-being with derived benchmarks. This screening-level tool was developed by EPA Region 2 to represent the environmental burden in a community in support of EJ evaluations, with links to census data via a GIS layer to support the demographic component of such assessments.	Similar to RAIMI but as a screening tool, focuses on inputs for Toxics Release Inventory (TRI) emissions, air toxics, and facility density. More detailed analyses of a community burden would be conducted at the local level.

(6.4) Cumulative Adjustment of Protective Concentration Levels (PCLs), TCEQ (Texas Commission on Environmental Quality); http://www.tceq.state.tx.us/comm_exec/ forms_pubs/pubs/rg/rg-366_trrp_18.html/view	PCLs are a set of toxicity-based screening criteria developed for use in risk assessments. Individual PCLs were derived to evaluate risks from individual chemicals, and TCEQ developed an equation to adjust these downward when evaluating multiple chemicals, when at least 10 carcinogenic or noncarcinogenic chemicals of concern (COCs) are identified for a given pathway. These adjustments reduce PCLs for individual chemicals based on the ratio of the measured concentration of each to its PCL. If the sum of these ratios exceeds a predetermined target, adjusted PCL values may be necessary for some COCs to ensure that state risk reduction rule mandates are met (e.g., to not exceed a risk of 10^−4^ or an HI of 10). COCs to be adjusted are determined from a decision process outlined in the guidance.	Can be used for screening calculations based on the sum of ratios approach (similar to NIOSH, IRSST, and others, including the approach used to assess radionuclides), under the default assumption of additivity.

(6.5) HEM-3, Human Exposure Model-3 (EPA); http://www.epa.gov/ttn/fera/hem_download.html	Designed to predict risks associated with chemicals released to ambient air, used primarily to assess risk for major point sources of air toxics. Generates results for one facility at a time focusing on the inhalation pathway. Contains an atmospheric dispersion model and U.S. census information at the census block level. Each source must be located by latitude and longitude, and its release parameters must be described. This tool is generally used to estimate concentrations within 50 km of a source. It provides ambient air concentrations as surrogates for lifetime exposure, for use with unit risk estimates and inhalation RfCs to produce estimates of cancer risk and noncancer HI, respectively.	Presents risk and noncancer estimates. To support a CRA, estimates for individual sources could be overlain to suggest insights for multiple sources.

(6.6) Probabilistic tools (Monte Carlo analysis resources), such as those described at: http://www.epa.gov/raf/prawhitepaper/index.htm; http://www.epa.gov/raf/prawhitepaper/index.htm	Statistical methods for addressing uncertainty and variability in estimating health risks by developing multiple descriptors to calculate a quantity repeatedly with randomly selected scenarios for each calculation. These are most useful for single-point risk estimates, and they can be used as a presentation tool because graphics show the range of scenarios and outputs.	Combining approximations for multiple sources of potential risk (e.g., from environment and lifestyle) is complicated. These tools could be used to combine results for individual exposures that consider variability and uncertainty.

(6.7) Software and User's Manual for the Integrated Exposure Uptake Biokinetic Model for Lead in Children (IEUBK) (also Adult Lead Model, other data) (EPA); http://www.epa.gov/superfund/lead/index.htm; http://www.epa.gov/superfund/lead/products.htm	The IEUBK model consists of four modules (exposure, uptake, biokinetic, and probability distribution) to estimate blood lead levels in children exposed to lead by various routes. A distribution of lead concentrations from the geometric mean can be used to estimate the risk that lead levels in blood for a child or group of children will exceed a target level. The tool is included here (in addition to the related entry in Table 4) because it can also be used to assess the uncertainty in the risk estimate.	Can estimate blood lead levels based on exposures to multiple sources via multiple routes using a complex set of variables that include adjustable exposure, uptake, and biokinetic parameters. (See related entry in Table 4.)

(6.8) Policy for Risk Characterization (EPA); http://www.epa.gov/OSA/spc/pdfs/rccover.pdf	Emphasizes transparency in decision making, clarity in communication, and consistency in assumptions and policies. Encourages plans that reflect these values and consistency and calls for programs to fall within a “zone of reasonableness.”	Encourages an open process as well as program- and region-specific policies, procedures, and implementation for CRAs.

(6.9) Elements to Consider When Drafting EPA Risk Characterizations; http://www.epa.gov/osa/spc/pdfs/rcelemen.pdf	Outlines the basic principles of risk characterization and presents an outline for developing chemical risk assessments that includes hazard identification, dose response, exposure, conclusions, and context.	Provides insights for applying risk characterization principles for CRAs, with suggestions for topics to consider when conducting an assessment.

(6.10) Handbook on Risk Characterization (EPA); http://www.epa.gov/spc/pdfs/rchandbk.pdf	Describes the importance of conducting the risk characterization process in a *transparent* manner, with products that are *clear*, *consistent*, and *reasonable* (TCCR). Appendices of this handbook include the EPA 1995 risk characterization policy and illustrative case studies.	The basic principles outlined in this report are useful for CRAs and can be helpful to risk assessment practitioners, managers, and the general public.
